# Choline sulfatase from *Ensifer* (*Sinorhizobium*) *meliloti*: Characterization of the unmodified enzyme

**DOI:** 10.1016/j.bbrep.2015.08.002

**Published:** 2015-08-07

**Authors:** Juan José Sánchez-Romero, Luis F. Olguin

**Affiliations:** ^a^Laboratorio de Biofisicoquímica, Facultad de Química, Universidad Nacional Autónoma de México, México D. F. 04510, México

**Keywords:** COS, *E. meliloti* choline-*O*-sulfatase, FGly, *α*-formylglycine, FGE, *α*-formylglycine-generating enzyme, anSME, anaerobic sulfatase maturing enzyme, pNPS, *p*-nitrophenyl sulfate, MUS, 4-methylumbelliferyl sulfate, ITC, isothermal titration calorimetry, DLS, dynamic light scattering, MALDI-TOF, matrix assisted laser desorption ionization time-of-flight, DTNB, 5,5′-Dithiobis(2-nitrobenzoic acid), DTT, DL-Dithiothreitol, TCEP, Tris(2-carboxyethyl)phosphine hydrochloride, UPLC-ESI-Q-TOF-MS, Ultra-performance liquid chromatography-electrospray ionization-quadrupole time-of-flight-mass spectrometry, Choline-*O*-sulfatase, Type I sulfatase, Formylglycine post-translational modification, Choline-*O*-sulfate, Catalytic efficiency

## Abstract

*Ensifer* (*Sinorhizobium*) *meliloti* is a nitrogen-fixing α-proteobacterium able to biosynthesize the osmoprotectant glycine betaine from choline sulfate through a metabolic pathway that starts with the enzyme choline-*O*-sulfatase. This protein seems to be widely distributed in microorganisms and thought to play an important role in their sulfur metabolism. However, only crude extracts with choline sulfatase activity have been studied. In this work, *Ensifer* (*Sinorhizobium*) *meliloti* choline-*O*-sulfatase was obtained in a high degree of purity after expression in *Escherichia coli*. Gel filtration and dynamic light scattering experiments showed that the recombinant enzyme exists as a dimer in solution. Using calorimetry, its catalytic activity against its natural substrate, choline-*O*-sulfate, gave a *k*_cat_=2.7×10^−1^ s^−1^ and a *K*_M_=11.1 mM. For the synthetic substrates *p*-nitrophenyl sulfate and methylumbelliferyl sulfate, the *k*_cat_ values were 3.5×10^−2^ s^−1^ and 4.3×10^−2^ s^−1^, with *K*_M_ values of 75.8 and 11.8 mM respectively. The low catalytic activity of the recombinant sulfatase was due to the absence of the formylglycine post-translational modification in its active-site cysteine 54. Nevertheless, unmodified *Ensifer* (*Sinorhizobium*) *meliloti* choline-*O*-sulfatase is a multiple-turnover enzyme with remarkable catalytic efficiency.

## Highlights

• First biochemical characterization of a recombinant choline-*O*-sulfatase.
• Recombinant enzyme has no post-translational modification in its active site cysteine.
• The unmodified enzyme exhibits multiple catalytic cycles.
• Despite a low *k*_cat_ the enzyme accelerate 10^20^-fold the uncatalyzed reaction.

## Graphical abstract

**Figure:**
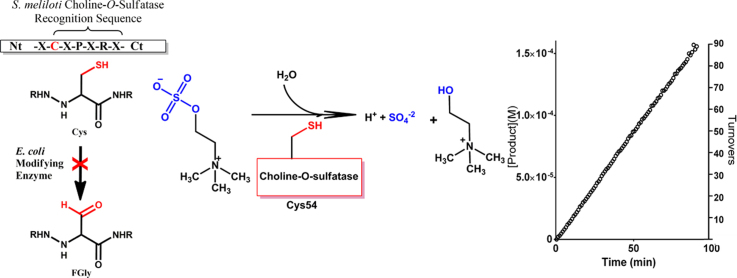

## 1 Introduction

**Fig. 1:**
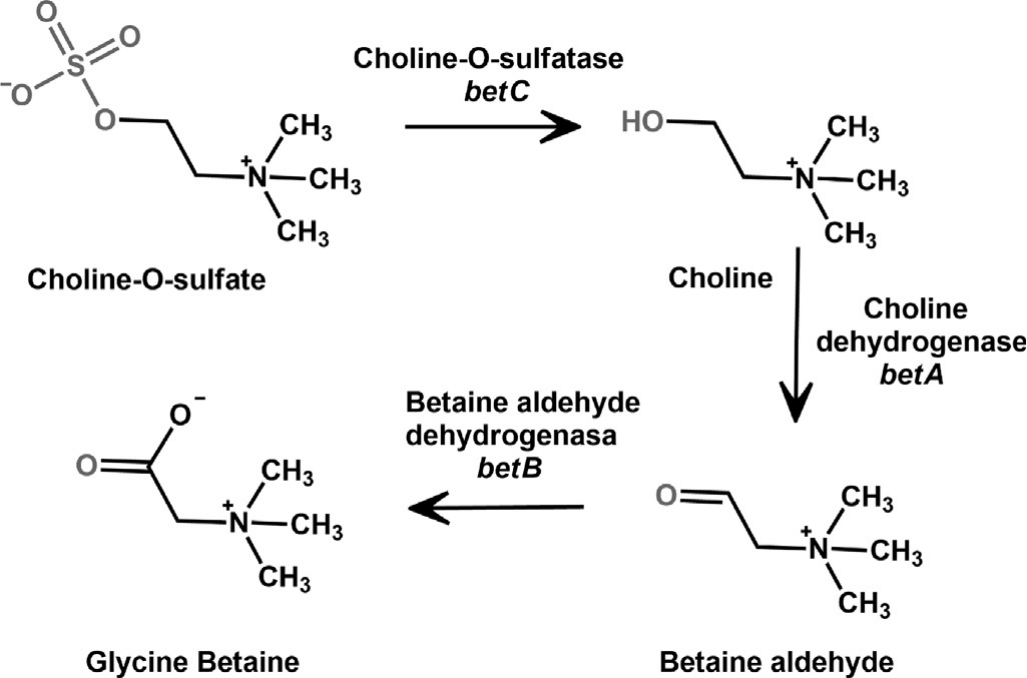
Synthesis of glycine betaine from choline sulfate in *E. meliloti*.

*Ensifer meliloti* (formerly *Sinorhizobium meliloti*) is a nitrogen-fixing α-proteobacterium that establishes root nodule symbiosis with legume plants*,* providing ammonia to their hosts and receiving nutrients from them [1]. In free life or in symbiosis, these bacteria have to deal with adverse environmental conditions such as droughts, rain or floods, which cause severe changes in their extracellular osmolality. An immediate response to cope with these situations is to accumulate or release ions and selected low-molecular-weight organic molecules called osmolytes that counteract the osmotic gradient [2], [3], [4], [5]. Glycine betaine is a potent and well-characterized osmoprotectant widespread in nature [5], [6], and *E. meliloti* can efficiently transport it to its interior through high-affinity uptake protein systems [4], [7]. Alternatively, it can synthesize glycine betaine from choline-*O*-sulfate by a three-step pathway with choline and betaine aldehyde as intermediates (Fig. 1) [8]. The genes involved in this metabolic pathway constitute the operon *betICBA*, which is composed of a regulatory gene (*betI*) and three structural genes: *betC* (choline-*O*-sulfatase, or COS), *betB* (betaine aldehyde dehydrogenase) and *betA* (choline dehydrogenase) [8].

The metabolic pathway is controlled by BetI, a repressor that regulates the expression of *bet* genes in response to the inducer choline [9]. Transcription of the operon can also be initiated to a lesser extent by the presence of choline-*O*-sulfate or acetylcholine, but not by the presence of high salt concentration alone [9]. However, as the product of the route, glycine betaine, is a potent osmolyte that accumulates in *E. meliloti* under salt stress [10], [11], choline-*O*-sulfate or choline can allow the proliferation of *E. meliloti* under high salt concentration through their transformation to glycine betaine [8], [9]. Under no salt stress, *E. meliloti* can import choline or choline-*O*-sulfate from its surroundings and transform them to glycine betaine, which can be further metabolized to cope entirely with the carbon and nitrogen cell demands in the absence of other nutrients [8], [11]. The inorganic sulfate produced by the hydrolysis of choline-*O*-sulfate can also be used as the only source of sulfur by *E. meliloti* [8]. Choline-*O*-sulfate, has been shown to be biosynthesized and accumulated by a variety of plants, marine and soil fungi and red algae where it serves as an osmoprotector and sulfur reservoir [12], [13], [14], [15], [16], [17], [18]. In contrast, certain bacteria, as *Bacillus subtilis, Escherichia coli and Salmonella typhimurium,* accumulate choline-*O*-sulfate as osmoprotector without further metabolization [19], [20]. Choline-*O*-sulfate can be released to the environment through root exudation or microbial cell decay [4], [21] and once in the soil, it can be taken by other microorganisms as an important source of sulfur, carbon and nitrogen [22], [23]. The extent of choline-*O*-sulfate in soil or other environments is not well characterized, however, its direct or indirect role as osmolyte and the widespread presence of choline sulfatase genes in microbes [24] suggest an important role of this compound and these enzymes in the biological sulfur cycle.

Amino acid sequence analysis of COS revealed that this enzyme belongs to the type I sulfatases family (previously named arylsulfatases) [8], [24]. These enzymes share high degree of conservation in sequence, structure and enzymatic mechanism among all life kingdoms and hydrolyze many diverse sulfate esters present in mono- oligo- and polysaccharides, proteoglycans, amino acids, steroids and glycolipids [25]. Recent genetic analyzes identified a few peptide signatures that seem to be specific for choline sulfatases [24]. In *E. meliloti* and other members of the *Rhizobiaceae* family, the gene *betC*, is in the operon *betICBA* mentioned above, but in the rest of microbes *betC* has a different genetic environment and is mainly found associated to an ABC-type betaine periplasmic binding protein and to an ATP-binding protein with a putative sulfate permease activity [22], [24]. All type I sulfatases, including COS, have a highly conserved amino acid sequence in their active site: (C/S)-X-(P/A)-X-R [25], [26], [27]. This sequence is critical, since it is the recognition site for a post-translational modification of an active site cysteine or serine residue, to the catalytically functional residue α-formylglycine (FGly). This modification is catalyzed by a formylglycine-generating enzyme (FGE), or by an anaerobic sulfatase maturing enzyme (anSME) depending on the organism [27]. *E. coli* is only able to modify cysteine residues and the identification of the enzymatic machinery responsible for this modification in this bacterium has been elusive [28].

Given the apparent ubiquity of choline-*O*-sulfatases in microorganisms [24], their study is important in understanding sulfur metabolism in microorganisms and soil, and in some contexts the osmoprotection capacity of their substrate. To our knowledge, there are only three reports of biochemical characterization of choline-*O*-sulfatases, but in all of them only crude extract or partially purified proteins were studied [14], [29], [30]. In this work the heterologous expression of COS was performed in *E. coli* BL21 (DE3), followed by complete purification and biochemical characterization.

## 2 Materials and methods

### 2.1 Molecular cloning and recombinant expression

COS gene (*betC*) sequence [AAC13371.1] was codon optimized for *E. coli* expression (Supplementary material Fig. S1), synthesized, and sequenced by GenScript USA Inc. Then, it was subcloned in pET26b+ with a C-terminus His_6_-tag sequence. Overexpression of COS was performed in *E. coli* BL21 (DE3) using IPTG (1.0 mM) at 30 °C for 7 h.

### 2.2 Enzyme purification

Cells were centrifuged and resuspended in 1/25 of their original volume in Tris–HCl buffer (200 mM) with imidazole (20 mM) pH 7.5. They were treated with lysozyme (1 mg/ml final concentration) for 30 min at 4 °C and lysed by sonication. The crude extract obtained after centrifugation was loaded into a His-Trap FF column (GE-Healthcare) and eluted with a linear gradient of 20–250 mM imidazole in 10-column volumes. Fractions were analyzed by 10% SDS-PAGE and those containing the enzyme were pooled and further purified by size exclusion chromatography (HiLoadSuperdex 200 16/600 GL; GE-Healthcare) using Tris–HCl (20 mM) buffer pH 7.5. Fractions containing the pure enzyme were mixed and its concentration calculated spectrophotometrically at 280 nm using a theoretical molar extinction coefficient of 97,750 M^−1^ cm^−1^ [31]. The enzyme was aliquoted and stored at −20 °C. Typical yields of purified protein were in the range 30–35 mg of protein/L of culture.

### 2.3 Biochemical characterization and enzymatic activity

The molecular weight of the native enzyme was determined with a gel filtration analytical column (Superdex 200 10/300 GL; GE-Healthcare) using as standards: thyroglobulin (670 kDa), gamma globulin (158 kDa), ovoalbumin (44 kDa), myoglobin (17 kDa) and vitamin B12 (1.35 kDa) (Gel Filtration Standard; Bio-Rad).

Masses were determined with a Bruker Microflex matrix assisted laser desorption ionization time-of-flight (MALDI TOF) instrument (Bruker Daltonics GmbH) equipped with a 20-Hz nitrogen laser at *λ*=337 nm. Spectra were recorded in reflector and/or linear positive mode for the mass range of 25,000–250,000 Da. 1.0 μL of sample solution was mixed with 5 μL of 30% acetonitrile, 70% water, 0.1% trifluoroacetic acid, and saturated with sinapinic acid. Then, 1.0 μL of this solution was deposited onto the MALDI target and allowed to dry at room temperature.

Dynamic light scattering (DLS) measurements were performed at 25.0 °C using a Malvern Nano S (Malvern, Ltd.) instrument equipped with laser NIBS (Non Invasive Back Scattering) technology and a Peltier temperature controller. The hydrodynamic radius was calculated using the Zeta Sizer software provided with the equipment.

Enzyme-catalyzed hydrolysis of choline-*O*-sulfate (Cambridge Isotope Laboratories) was measured by Isothermal Titration Calorimetry (ITC) using a VP-ITC microcalorimeter (Microcal Inc.) at 25 °C in the Reaction Buffer (200 mM Tris–HCl pH 7.5 and 500 mM NaCl). The kinetic parameters were obtained following the procedure of multiple injections [32] in which a COS solution (2.46 µM) was incubated in the calorimetric cell and a solution of choline-*O*-sulfate (300 mM) was injected multiple times (2×5 µL and then 18×10 µL) to acquire a calorimetric thermogram. The calorimetric data were transformed to initial rate vs substrate concentration plots using the ITC Data Analysis in Origin 7.0 (Microcal Inc.) [32]. The enthalpy of the reaction was determined by triplicate using the single injection method in which a COS solution (21.4 µM) was incubated in the calorimetric cell and a single injection (1×10 µL) of choline-*O*-sulfate (300 mM) was done. The kinetic constants *k*_cat_, *K*_m_ and *K*_i_ were calculated from nonlinear least square fitting of the initial rate vs substrate concentration plots using the GraphPad Prism 6 software. Fundaments of the calorimetry technique to obtain kinetic parameters have been fully illustrated [32], [33], [34].

COS enzymatic activity against *p*-nitrophenyl sulfate (pNPS, Sigma-Aldrich) or 4-methylumbelliferyl sulfate (MUS, Sigma-Aldrich) was measured in the Reaction Buffer at 25 °C. Substrate concentrations were varied between 1 and 80 mM or 1 and 20 mM respectively, using a constant enzyme concentration of 1.7 µM. The amount of *p*-nitrophenol or methylumbelliferone released by COS activity was determined by measuring the absorbance changes at 400 nm or 370 nm respectively with a Cary-Bio50 (Varian/Agilent) spectrophotometer.

The pH dependence of COS enzymatic activity was studied using pNPS as substrate. Buffer solutions were sodium acetate/acetic acid (200 mM) and NaCl (500 mM) for pH 5.5 and Tris–HCl (200 mM) and NaCl (500 mM) for pH 6.5–8.5. The hydrolysis rate was determined spectrophotometrically at 400 nm and 25 °C. For pH 5.5, aliquots of the reaction mixture were taken at different times, adjusted to pH 8.0 with 1 M Tris–HCl and its absorbance immediately measured at 400 nm.

Product inhibition by choline was measured at 25 °C using as substrate pNPS in the Reaction Buffer, with 1.7 µM of enzyme and pNPS in a concentration of 1–80 mM. Choline chloride (Sigma-Aldrich) was added as an inhibitor at concentration of 20, 50 and 70 mM. The activity was obtained by measuring the absorbance changes at 400 nm.

### 2.4 FGly determination

The total number of cysteines present in a sample of COS was determined by the Ellman's reaction [35], [36]. A 2 mM stock solution of 5,5′-Dithiobis(2-nitrobenzoic acid) (DTNB, Sigma-Aldrich) in 50 mM sodium acetate was prepared, and 50 µL of this DTNB stock solution and 100 µL of 1 M Tris–HCl buffer pH 8.0 were mixed with distilled water up to a volume of 990 µL. Then, 10 µL of one of the following protein solutions (250 µM) were added: 1) native protein; 2) protein previously treated with 8 M urea; 3) protein treated with 8 M urea and 100 mM DL-Dithiothreitol (DTT, Sigma-Aldrich) and 4) protein treated with 8 M urea and 100 mM Tris(2-carboxyethyl)phosphine hydrochloride (TCEP, Sigma-Aldrich). Remaining DTT and TCEP were removed from samples 3 and 4 before mixing them with the DTNB solution. This was done by buffer exchange using an ultrafiltration device (Vivaspin 20, 10,000 MWCO, Sartorius) employing an 8 M urea solution. The reaction mixtures with DTNB were incubated at room temperature for 5 min and the changes in absorbance at 412 nm were measured. Cysteine concentration was determined by comparison to a standard curve obtained with acetyl cysteine and DTNB (molar extinction coefficient of 13,689±122.9 M^−1^ cm^−1^).

Additionally, the absence of FGly in a COS sample was investigated by ultra-performance liquid chromatography coupled to electrospray ionization tandem quadrupole time-of-flight mass spectrometry (UPLC-ESI-Q-TOF-MS) based on the procedure described by Rabuka et al. [37]. After purification, ~200 µg of COS were denatured in 6.0 M urea and 100 mM Tris pH 8.5 for 15 min. The sample was then incubated with DTT (50 mM) at 37 °C for 45 min, followed by alkylation with iodoacetamide (30 mM) for 60 min. After purification, the sample was digested with porcine trypsin for 18 h at 37 °C. It was then desalted and concentrated in a Ziptip C18 column, using 12 µL of 3% acetonitrile and 0.1% formic acid in water as the mobile phase. The eluted sample was analyzed by UPLC-ESI-QTOF-MS (nanoACQUITY-Waters – SYNAPT G2S Waters).

## 3 Results

### 3.1 Expression and biochemical characterization of COS

**Fig. 2:**
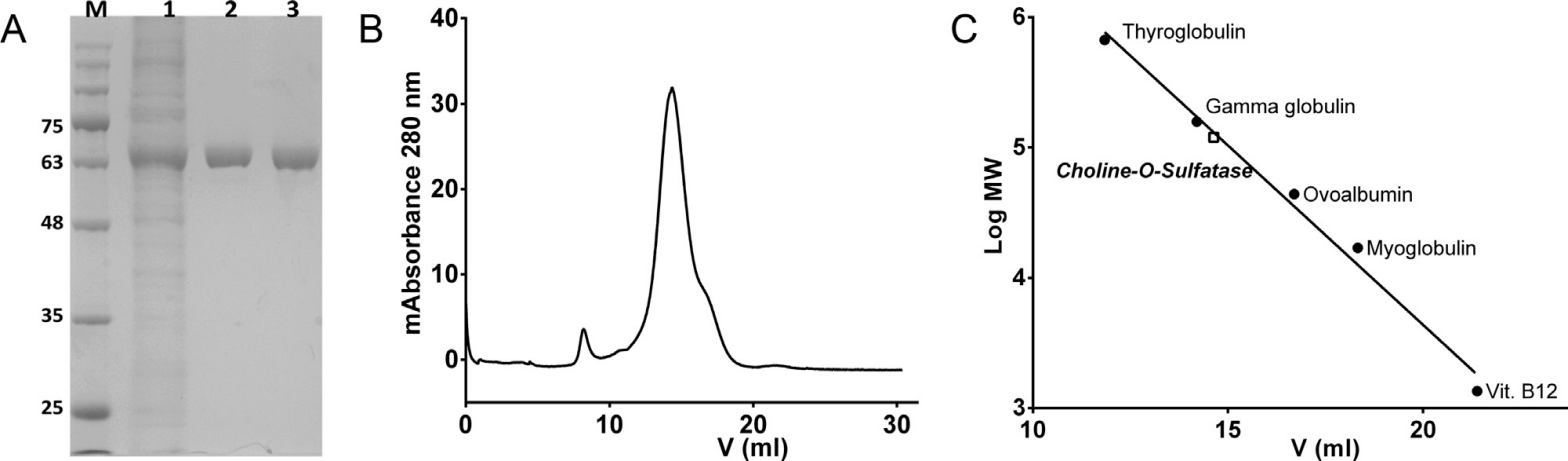
(A) SDS-PAGE of the purification of COS. *Lane* 1 crude extract; *lane* 2 fractions eluted from the His-Trap column containing COS; *lane* 3 fractions eluted from the gel filtration column containing COS. (B) Molecular exclusion chromatography of COS on a Superdex 200 column. (C) Determination of the molecular mass of COS by molecular exclusion chromatography.

The choline-*O*-sulfatase gen from *E. meliloti* was efficiently expressed in *E. coli* BL21 (DE3) after induction with 1.0 mM IPTG for 7 h at 30 °C (Fig. 2A; lane 1). Its purification was easily achieved thanks to a His_6_-Tag at its C-terminus (Fig. 2A; lane 2) and a final purification step through a gel filtration column (Fig. 2A; lane 3) was performed to remove several soluble aggregates of protein not visible in the SDS-PAGE gel (Fig. 2B).

The apparent molecular mass of COS was ~63 kDa as estimated by SDS-PAGE (Fig. 2A), which is in agreement with the expected 59 kDa value considering the His_6_-Tag. Subsequently, the molecular mass was determined in non-denaturing conditions using a gel filtration analytical column giving a molecular mass of 123 kDa (Fig. 2C), indicating that COS exists in a dimeric form in solution. DLS tests exhibited a single peak with a hydrodynamic radius of 5.6 nm consistent with a dimeric form of the protein (Supplementary material Fig. S2). The sample was also subjected to MALDI-TOF mass spectrometry giving two peaks at 118 and 59 kDa corresponding to the dimeric and monomeric forms of COS (Supplementary material Fig. S3).

### 3.2 Kinetic characterization of COS

**Fig. 3:**
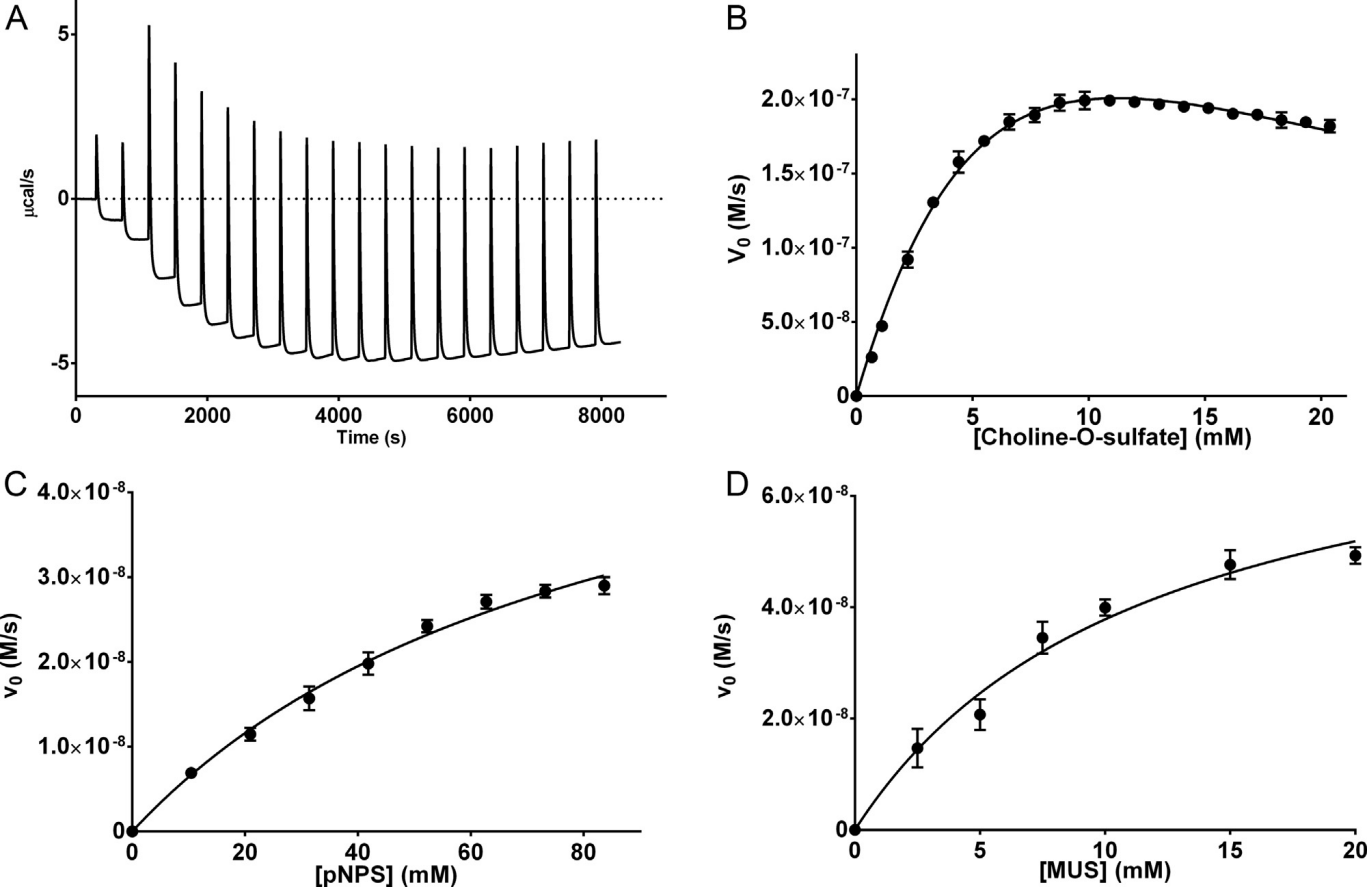
Determination of COS kinetic parameters at pH 7.5 and 25 °C in 200 mM Tris–HCl and 500 mM NaCl. (A) Calorimetric initial rate measurements. The figure shows a typical thermogram obtained by ITC in which a COS solution (2.46 µM) was titrated with choline-*O*-sulfate (300 mM) (2×5 µL and then 18×10 µL). Each titration was done before a significant amount of choline-*O*-sulfate was hydrolyzed. (B) Michaelis–Menten plot for the choline-*O*-sulfate hydrolysis. Data was obtained by transforming the calorimetric data to initial rates using procedures reported in the literature (see Section 2). (C) and (D) Michaelis–Menten plot for the hydrolysis of pNPS and MUS respectively obtained by UV–vis spectrophotometry.

**Fig. 4:**
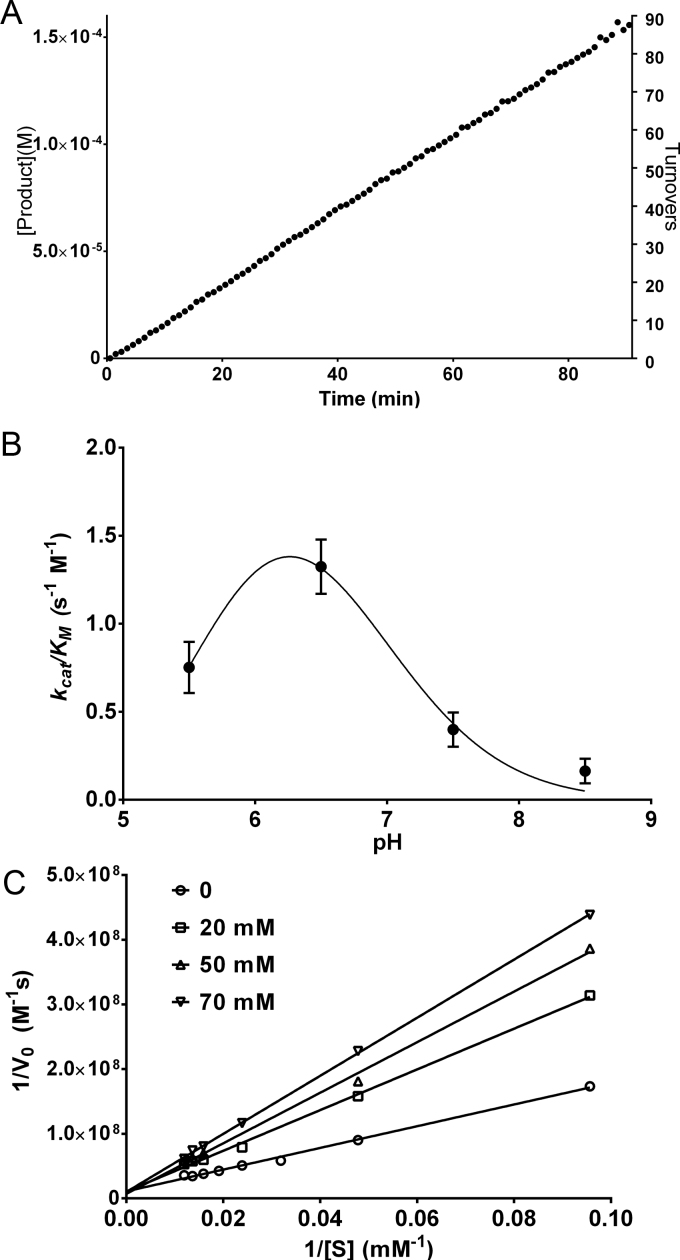
(A) Time course of the COS catalyzed hydrolysis of pNPS at pH 7.5 and 25 °C; [COS]=1.74×10^−6^ M and [pNPS]=70 mM. (B) COS catalyzed hydrolysis of pNPS as a function of pH at 25 °C. (C) Competitive inhibition of COS by choline at 25 °C and pH 7.5.

COS-catalyzed hydrolysis of choline-*O*-sulfate was measured by calorimetry since non-appreciable change in the UV–vis spectra is observable for this reaction. Multiple injections of the substrate to a calorimetric cell with the enzyme gave a typical thermogram (Fig. 3A) that was interpreted and transformed to a usual Michaelis–Menten plot (Fig. 3B) [32]. The observed peaks in the thermogram correspond to the dilution heat of the substrate reaching the calorimetric cell. After each peak the thermal power returns to a steady-state level that corresponds to the initial rate at that substrate concentration. Before significant substrate depletion occurs, a subsequent injection is made to obtain a different initial rate corresponding to a new higher substrate concentration. This process is repeated multiple times to obtain the thermogram. The more negative steady-state level observed after each injection corresponded to a higher rate. However, at ~4000 s the thermal power started to increase (lower rate) indicating the presence of substrate inhibition (Fig. 3A and B). To transform the calorimetric data from the thermogram to enzymatic initial rates it is necessary to experimentally determine the apparent reaction enthalpy (∆*H*_app_). This was obtained by a single injection experiment (Supplementary material Fig. S4), giving a ∆*H*_app_=−73.63±3.34 kJ/mol (−17,600±800 cal/mol). Table 1 shows the kinetic parameters for the COS catalyzed choline-*O*-sulfate hydrolysis together with those obtained by spectrophotometry for the synthetic substrates pNPS and MUS. These last two substrates did not show enzyme saturation at their highest solubility in the buffer employed (Fig. 3C and D). Despite the low activity observed for COS, multiple catalytic cycles were observed for the substrates. For example, for pNPS we measured 73 turnovers per hour (Fig. 4A), while for choline-O-sulfate 93 turnovers were measured in the same period of time in a single injection experiment (Supplementary material Fig. S4). Additionally, COS activity against choline phosphate was tested by calorimetry, but no activity was detected, even though a 20-fold more enzyme than with choline-*O*-sulfate was used (Supplementary material Fig. S5). This contrasts with the reported by Osteras et al. [8], who observed phosphatase activity towards choline phosphate in *E. meliloti* crude extract obtained after COS induction.

**Table 1:** Kinetic parameters for COS catalyzed hydrolysis of sulfate esters substrates at 25 °C and pH 7.5. Reported values represent the average of three independent measurements and error ranges represent one standard deviation.

**Substrate**	***k*_cat_ (s**^−1^**)**	***K*_M_ (mM)**	***K*_i_ (mM)**^a^	***k*_cat_**/***K*_M_ (s**^−1^ **M**^−1^**)**
**Choline-*****O*****-sulfate**	0.27±0.02	11.1±1.0	11.4 ±1.1	24.32±2.84
**pNPS**	0.035±0.004	75.8±15.0	–	0.46±0.11
**MUS**	0.043±0.007	11.8±1.4	–	3.64±0.73

a Substrate inhibition constant.

### 3.3 COS activity-pH profiles and product inhibition

The enzymatic activity of COS was tested in the pH range 5.5–8.5 using pNPS as the substrate. We observed a bell shaped profile of COS specificity constant (*k*_cat_/*K*_M_) showing a maximum at pH 6.5 (Fig. 4C). Individual graphs of *k*_cat_ and *K*_M_ as a function of pH are shown in Supplementary material Figs. S6 and S7.

The possible inhibitory effects of the cognate reaction products, choline and inorganic sulfate, in COS catalyzed hydrolysis were examined using pNPS as substrate. Choline inhibited COS competitively with an inhibition constant (*K*_i_) of 57.8 mM (Fig. 4D) but no inhibition was observed with ammonium sulfate up to a 70 mM concentration.

### 3.4 Determination of FGly modification

Due to the low COS catalytic activity, the amount of post-translational modification of the cysteine 54, which is in the sulfatase recognition sequence C-X-P-X-R, was evaluated. First, a quantification of the total number of cysteines per mole of protein was performed using the Ellman's reaction. From the six possible cysteines present in the amino acid sequence, all of them were detected, suggesting the complete absence of the FGly residue (Table 2). Two of these cysteines were forming a disulfide bridge (compare samples treated with urea and urea+DTT or TCEP in Table 2).

**Table 2:** Quantification of cysteines present in COS using the Ellman's reaction. Values are the average of three independent determinations with their corresponding standard deviations.

**Treatment of protein sample**	**Number of detected Cys per mole of protein**
**None (native protein)**	0.30±0.01
**Urea 8 M**	3.99±0.03
**Urea 8 M+DTT**	6.05±0.09
**Urea 8 M+TCEP**	5.98±0.01

**Fig. 5:**
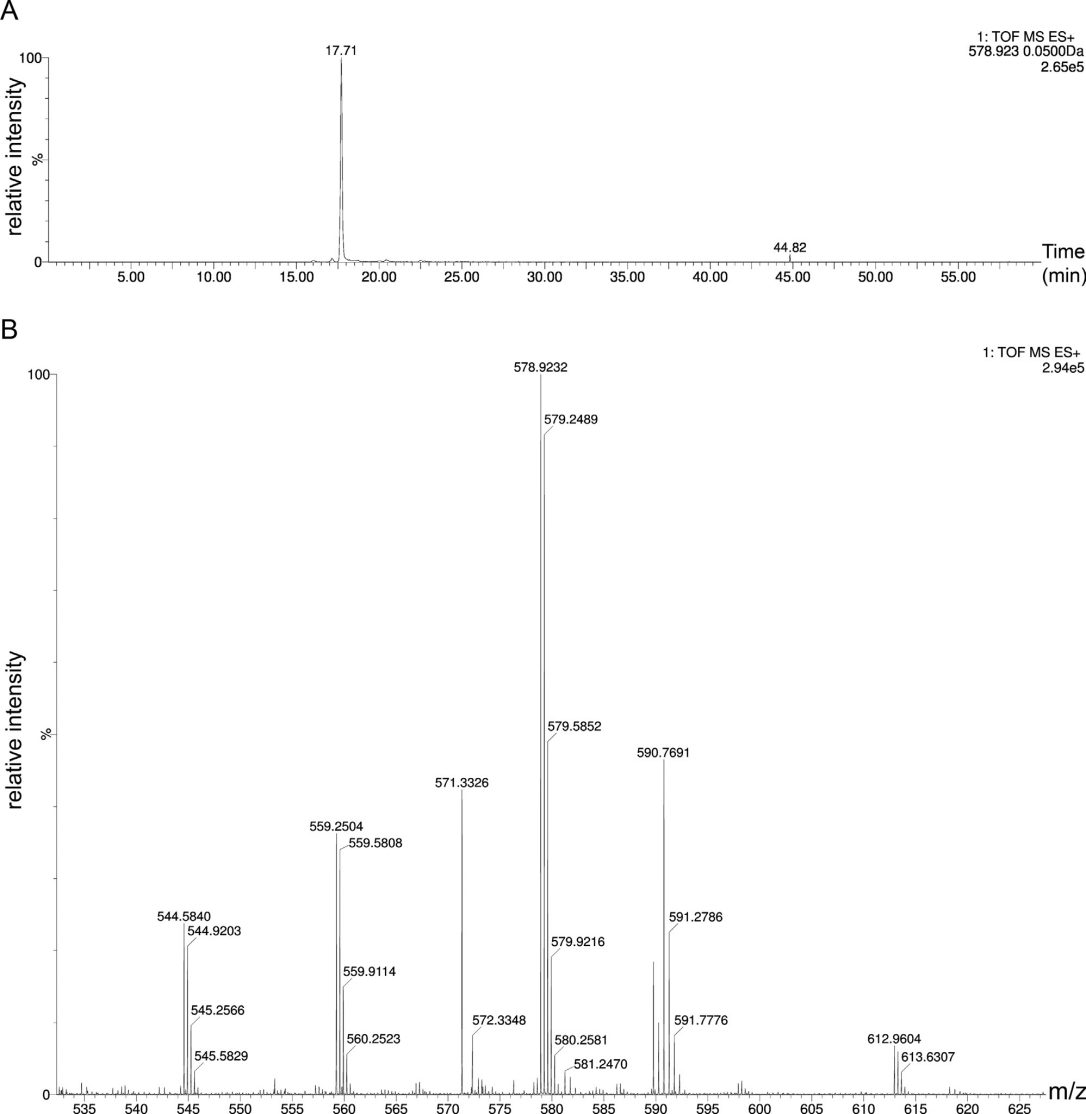
Results of UPLC-ESI-Q-TOF-MS analysis for COS. The protein was reduced with DTT, alkylated with iodoacetamide, digested with porcine trypsin and analyzed by LC–MS. (A) Extracted ion currents (XIC) at *m/z*=578.9232 corresponding to the peptide FHNNYTSSPL**C**APAR [M+3H]^+3^ with the carbamidomethylated Cys54. (B) Mass spectra of the same peptide (theoretical weight=1734.8016 Da and [M+3H]^+3^=578.9387).

To confirm the absence of the FGly modification in the cysteine 54, an UPLC-ESI-Q-TOF-MS analysis of a reduced, alkylated and trypsin-digested sample of COS was carried out using a established procedure from the literature [37]. The results showed a coverage of 89.5% of the protein sequence, including all cysteine-containing peptides. Amongst them was a peptide eluted at 17.71 min with a *m/z*=578.9232 corresponding to the triply protonated state [M+3H]^3+^ of the peptide containing the Cys54 in the carbamidomethylated-form (Fig. 5A and B).The FGly residue is an aldehyde that hydrates in water to give a geminol diol. In MS, both, the peptide with aldehyde and the one with the diol have been observed with two distinct molecular masses [37]. The expected COS FGly peptides (aldehyde M^+3H^=553.9390 *m/z* or diol M^+3H^=559.9425 *m/z*) with the same or different protonation state as the Cys containing peptide were extensively searched but were not detected in the MS analysis (for two independent determinations). These results confirm the absence of the FGly residue when COS is expressed in the conditions mentioned here.

## 4 Discussion

Recombinant *E. meliloti* COS was overexpressed and purified in high yields from *E. coli* cultures. However, its catalytic activity was poor as a result of the complete absence of the FGly post-translational modification in its active site. This lack of modification occurs despite COS sequence having the “sulfatase signature” (**C**-X-P-X-R) [25], [26], [27]. Table 3 shows that this consensus sequence can be recognized and transformed to FGly in different degrees in *E. coli* by its enzymatic machinery, which has not been identified yet [28]. In some cases, a mixture of unmodified cysteine and FGly has been observed by qualitative MALDI-TOF analysis, while in others only the peptide with the FGly has been detected indicating 100% of modification. Only in three examples the degree of FGly formation has been directly quantified in mixtures with the unmodified Cys peptides. For about half of all cases, the percentage of FGly has not been determined. Based on these results, it is clear that the presence of the minimum five “sulfatase signature” residues, or even the extended sequence depicted in Table 3, is not a guarantee of even partially FGly modification in *E. coli*. Human iduronate 2-sulfate sulfatase and COS have a serine residue at position (−13) with respect to the active site cysteine that is different from the consensus glycine residue, but still with glycine at this position the modification can be low as it was the case with *Mycobacterium tuberculosis* sulfatase [38].

**Table 3:** Recognition sequences for the modification of the active site cysteine to FGly in enzymes expressed in *E. coli*, and the percentage of FGly transformation.

**Type I sulfatase**	**Sequence**	**FGly presence**	**Method for FGly determination**	**Enzyme co-expressed**	**Ref.**
*F. heparinum*	(69)**G**TRFTRAYCAQPL**C**T**P**S**R**SAIFS**G**	100%	MALDI-MS		[39]
*FH2S*					
*C. perfringens*	(37)**G**YNFENAYTAVPS**C**I**A**S**R**ASILT**G**	Partially	MALDI-TOF	–	[40]
(BAB79937.1)		100%		anSME	
*P. aeruginosa*	(39)**G**LRLTDFHTAST-**C**S**P**T**R**SMLLT**G**	95%	DTNB	–	[41]
(CAA88421.2)					
*E. meliloti*	(41)**S**ARFHNNYTSSPL**C**A**P**A**R**ASFMA**G**	0%	DTNB	–	This work
(AAC13371.1)					
*M. tuberculosis*	(45)**G**ILFTRAHATAPL**C**T**P**S**R**GSLFT**G**	ND^a^	–	–	[38]
(F70837)					
*H. sapiens*	(66)**G**LLFPNFYSANPL**C**S**P**S**R**AALLT**G**	ND^a^	–	–	[42]
*(NP_000503.1)*					
*H. sapiens*	(71)**S**LLFQNAFAQQAV**C**A**P**S**R**VSFLT**G**	ND^a^	–	–	[43]
*(AAC77828.1)*					
*P. sp* ATCC19151 (CBI83290.1)	(40)**G**VVFDSAYCNSPL**C**A**P**S**R**FTLVS**G**	ND	–	–	[23]
*F. heparinum*	(67)**G**MLFNNCFVTNAV**C**G**P**S**R**ATILT**G**	ND	–	–	[44]
*FH6S*					
*F. heparinum*	(67)**G**VRFTNAFCSSPS**C**T**P**A**R**AGMLT**G**	ND	–	–	[45]
*NSulf*					
*H. pomatia*	(71)**G**VRLENYYVQ-PI**C**T**P**T**R**SQLMS**G**	b	–	–	[46]
(AAF30402.1)					
**Phosphonate monoester hydrolase**					
			
*R.leguminosarum*	(49)**G**TLFRRHYAGAAP**C**S**P**A**R**ATLYT**G**	25%	Fluorophore labeling	–	[47]
(WP025417352.1)		62%		*Mtb*FGE	
*B. caryophylli*	(45)**G**LTFRNHVTTCVP**C**G**P**A**R**ASLLT**G**	Partially	MALDI-TOF	–	[48]
(AAC44467.1)		Partially^c^		*Mtb*FGE	

ND: Not determined.

a Low activity suggest none or poor FGly modification.

b Expression in *E. coli* was not successful.

c Partially, but greater than without *Mtb*FGE.

To increase the FGly modification in recombinant *Clostridium perfringens* sulfatase, coexpression with its corresponding anSME in *E. coli* was done [40]. Similarly, phosphonate monoester hydrolases (PMHs), which are enzymes with the sulfatase recognition sequence, had been coexpressed with *M. tuberculosis* FGE (*Mtb*FGE) to increase the FGly formation [47], [48]. This last method efficiently (>85%) modifies N- or C-terminus FGE recognition sequences in some proteins [37], [40], [47], but in PMHs the conversions were lower [47], [48]. *E. meliloti* genome includes a putative FGE and its coexpression with COS will be tested in the near future in our group. In *E. meliloti* cells, the presence of the FGE should ensure that the modification occurs, obtaining a more active COS. Further studies that elucidate the sequence and/or structural requirements for expression of fully FGly-modified type I sulfatases in *E. coli* would be very beneficial for scientific and biotechnological purposes.

Despite the absence of the post-translational modification, COS was a competent enzyme able to perform multiple catalytic cycles. Another example of an enzyme lacking completely the FGly modification but still with hydrolytic activity is *Burkholderia caryophylli* phosphonate monoester hydrolase (BCPMH) [48]. Despite this, it would be expected that the presence of FGly in COS would increase the enzymatic activity, especially the *k*_cat_, by 5 to 2500-fold as it was observed for BCPMH and other sulfatases [41], [47], [48], [49]. In those examples, a comparison of the unmodified and modified enzyme was possible, or a Cys to Ser mutation in their active sites was done to prevent FGly formation in *E. coli.* In contrast, the 11.1 mM *K*_M_ value obtained in this work for COS is likely to be conserved for the enzyme carrying the FGly, as it was the case in all partially modified or Cys to Ser mutants [41], [47], [48], [49]. This relatively high *K*_M_ value is consistent with other *K*_M_s for choline sulfatase from natural sources: 40 mM for *Pseudomonas nitroreducens* [29]; 35 mM for *Aspergillus nidulans* [14] and 1.4 mM for *Pseudomonas aeruginose* [30], and could imply that the intracellular concentrations of choline-*O*-sulfate are in the millimolar range, like it is for many other osmoprotectants [2], [10]. Also the substrate inhibition observed for COS, might be used to prevent the fast depletion of choline-*O*-sulfate when it is in high concentration [50] and preserve its osmoprotectant effect.

Regarding its substrate specificity, COS showed an ~8-fold lower *k*_cat_ for the aromatic sulfate ester pNPS, compared to the alkyl choline-O-sulfate ester, despite the first one being ~10^8^-fold more labile [51]. This behavior is contrary to other type I sulfatases that show higher activities and lower *K*_M_ values for the aromatic substrates [25]. This low or null activity towards aromatic sulfates could be a common characteristic of choline sulfatases as it was also observed for *P. nitroreducens* choline sulfatase [29].

Even with the low *k*_cat_ values obtained for the unmodified COS, this enzyme has a remarkable catalytic proficiency as it would be accelerating the reaction 10^20^-fold compared to the uncatalyzed reaction (*k*_uncat_=2.25×10^−21^ s^−1^ estimated by using the equation of the Brønsted plot reported [51] and using a p*K*_a_=13.9 for choline sulfate [52]).

## 5 Conclusions

COS is a dimeric protein that can be expressed in high yields in *E. coli,* but under the conditions described here, it lacks completely the FGly maturation despite having the **C**-X-P-X-R sulfatase signature. Regardless of this, COS is able to hydrolyze its natural substrate choline-*O*-sulfate through multiple catalytic turnovers and with remarkable proficiency. Given the apparent ubiquity of choline sulfatases in microorganisms, further studies on their mechanism of action, the sequence or structural requirements for their post-translational modification and their substrate specificity would be important to understand the role of choline sulfatases in microbial sulfur metabolism and in the biological sulfur cycle.

## Footnotes

Appendix A Supplementary data associated with this article can be found in the online version at http://doi:10.1016/j.bbrep.2015.08.002.
