# Supplementary material for: Choline sulfatase from Ensifer (Sinorhizobium) meliloti: Characterization of the unmodified enzyme
Source: Biochem Biophys Rep. 2015 Aug 7;3:161–8. doi: 10.1016/j.bbrep.2015.08.002 (PMC6189696; doi:10.1016/j.bbrep.2015.08.002)
Supplement: Supplementary file 1 — Supplementary material [file mmc1.doc]

Supplementary material for:

Choline Sulfatase from *Ensifer (Sinorhizobium) meliloti*: Characterization of the Unmodified Enzyme

Juan José Sánchez-Romero^a^ and Luis F. Olguin^a^*

*^a^Laboratorio de Biofisicoquímica, Facultad de Química,*

*Universidad Nacional Autónoma de México, México D. F. 04510*

**Corresponding author*

*E-mail address:* [*olguin.lf@comunidad.unam.mx*](mailto:adam.abate@ucsf.edu)*;*

*Tel: +52 55 56223899 Ext 44434*


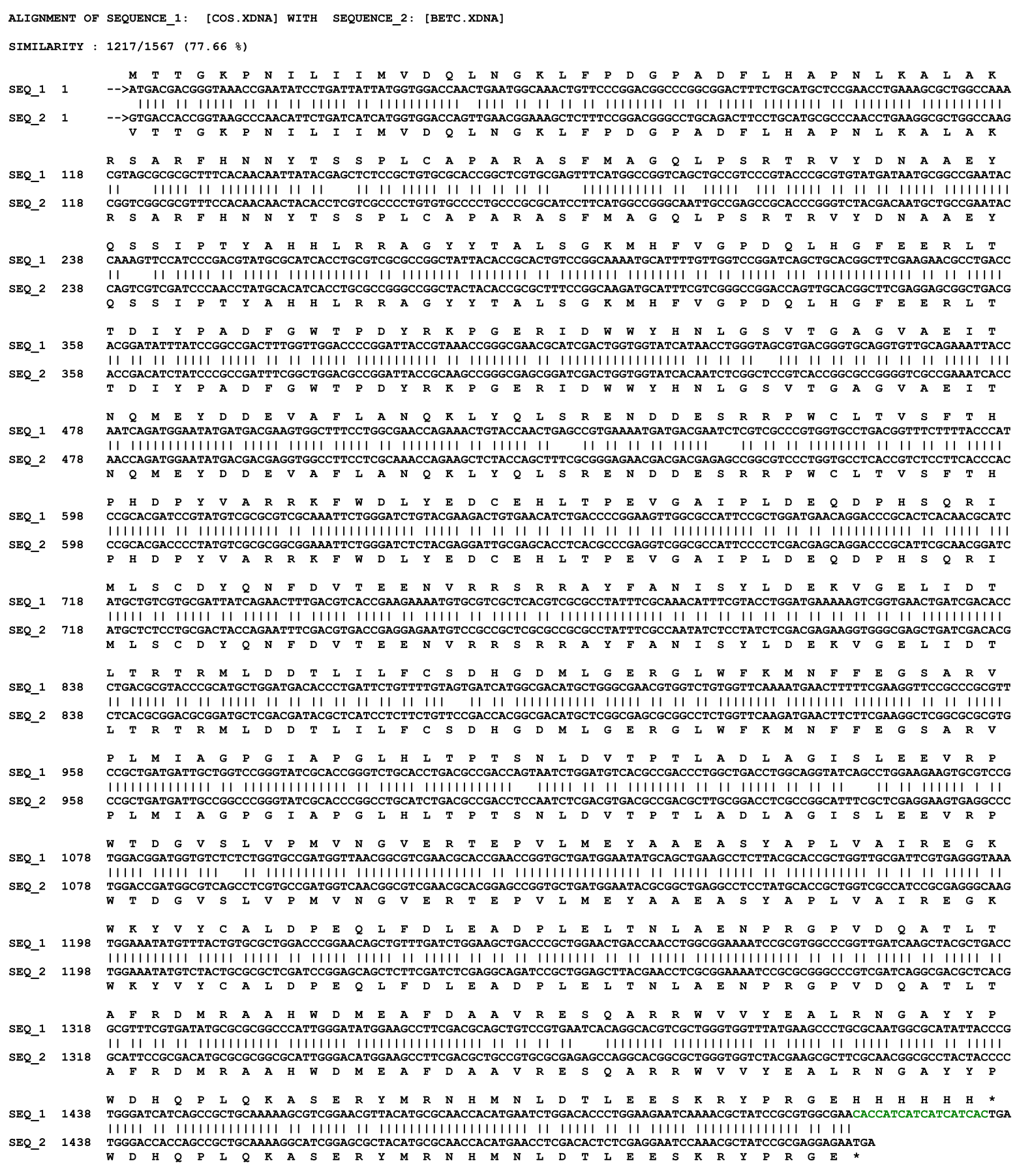


**Fig. S1.** Comparison between the wild type COS gen (*betC*) sequence (SEQ_2) with the codon optimized COS gene for *E. coli* expression (SEQ_1).


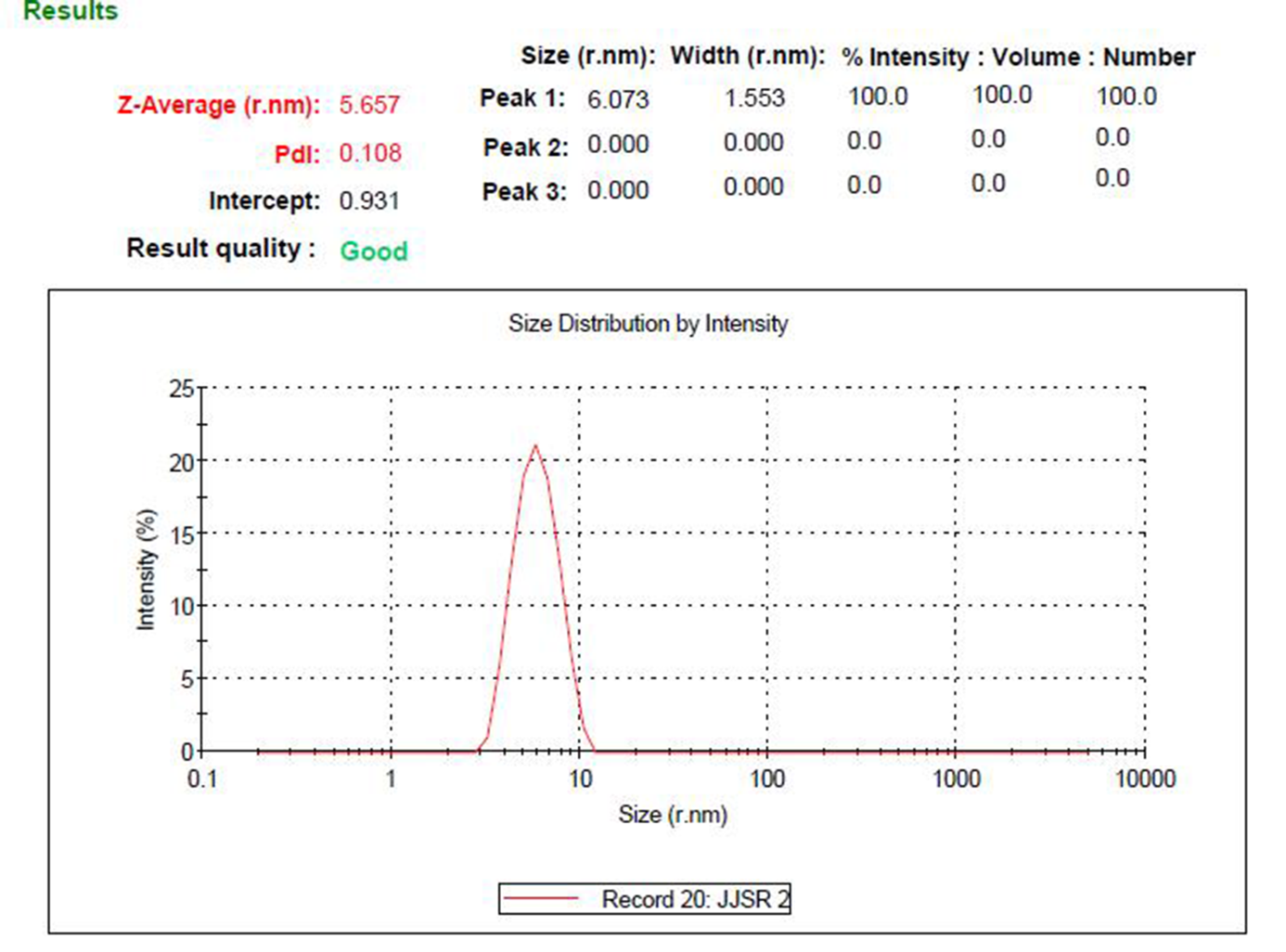


**Fig. S2.** DLS analysis results of a purified COS sample. A single peak with a hydrodynamic radius of 5.6 nm is shown.


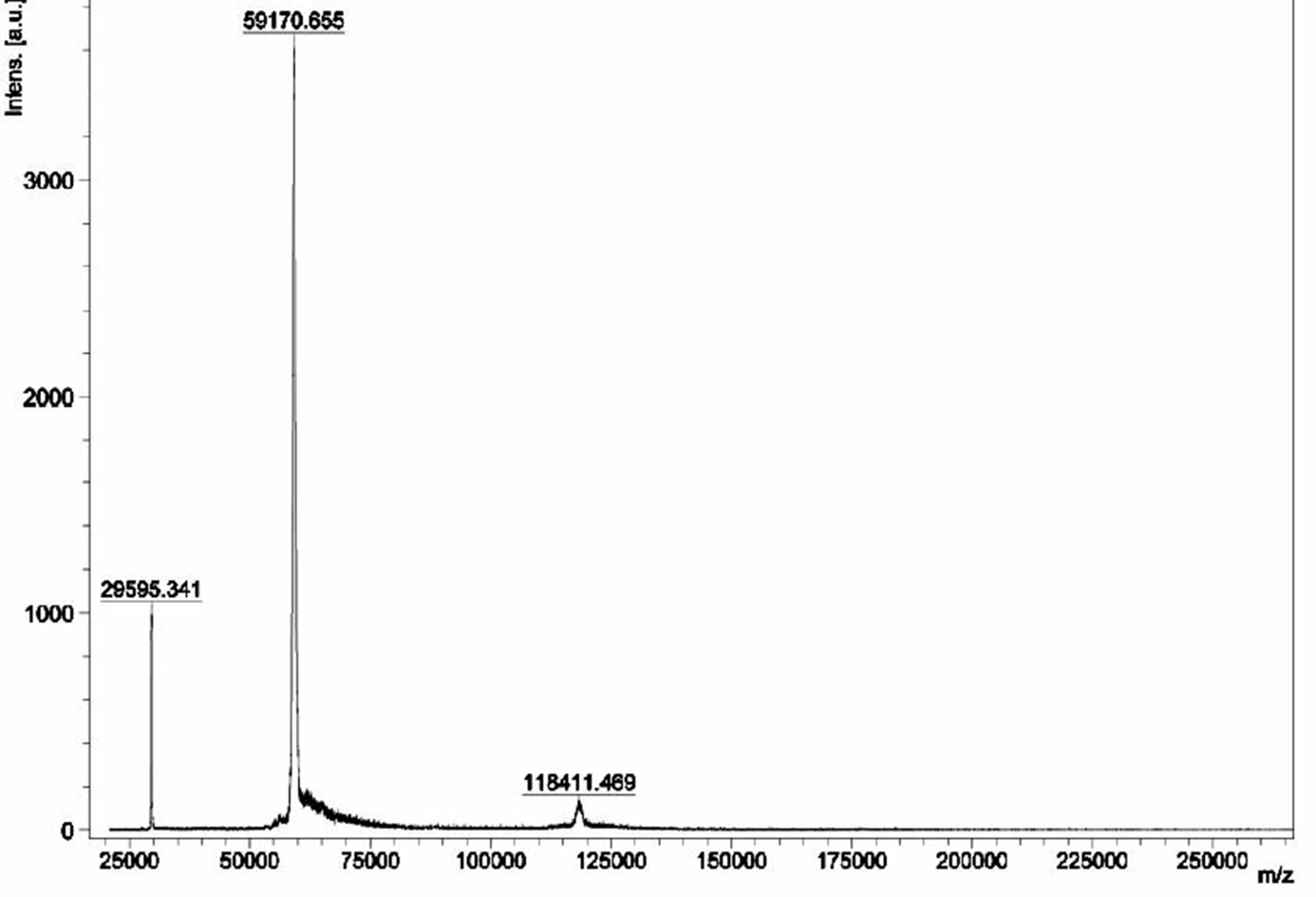


**Fig. S3.** MALDI-TOF results for COS. Two peaks appear corresponding to the monomer (59 kDa) and dimer (118 kDa) forms of the protein.


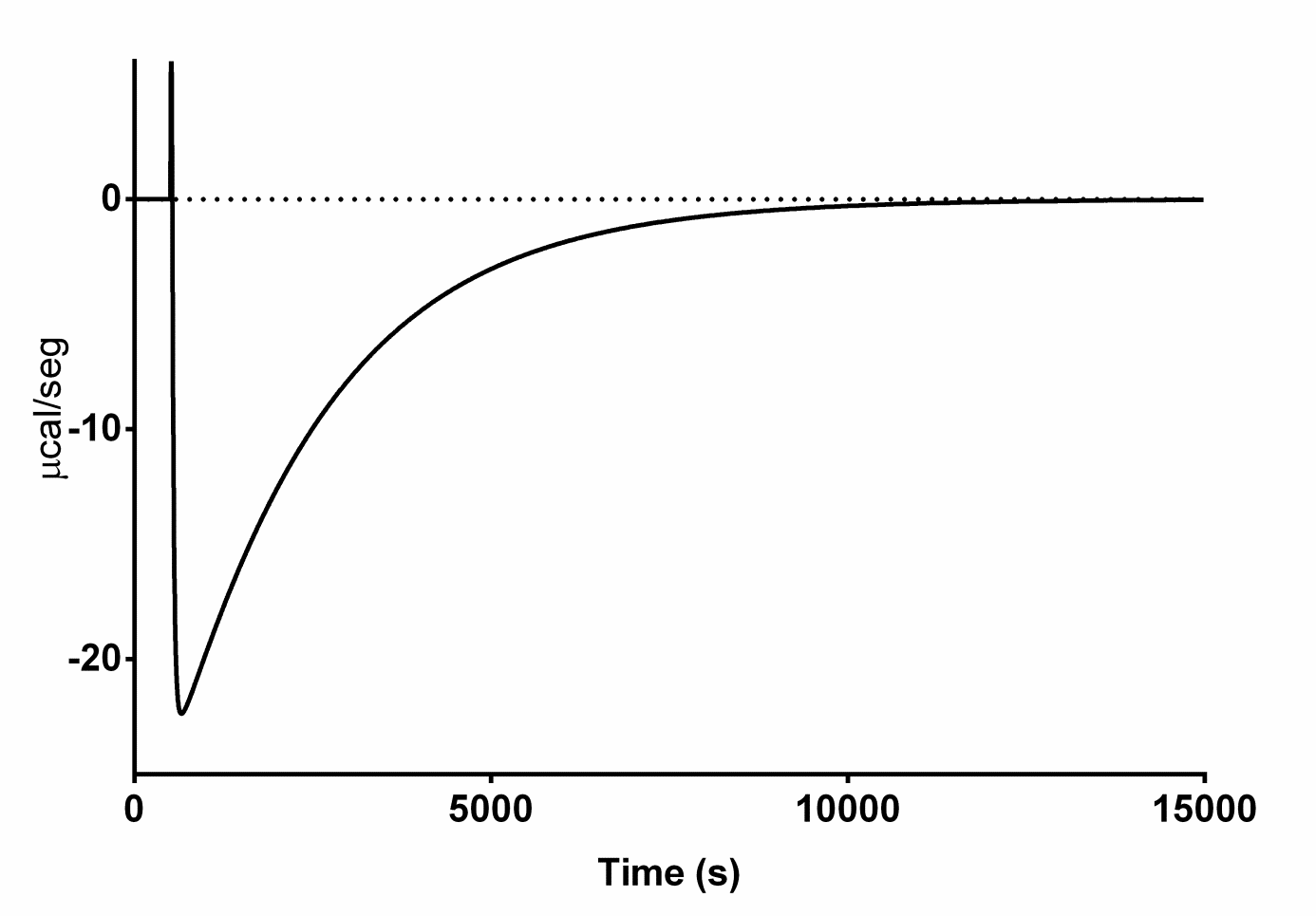


**Fig. S4.** The apparent enthalpy (∆*H*_app_) of the enzymatic hydrolysis of choline-*O*-sulfate by COS was determined by ITC using the single injection method. A COS solution (21.4 µM) was incubated in the calorimetric cell (1.420 mL) and a single injection (1 x 10 µL) of choline-*O*-sulfate (300 mM) was done. The average value of three independent measurements with their standard deviation are ∆*H*_app_ = ‑73.63 ± 3.34 kJ/mol (-17,600 ± 800 cal/mol).


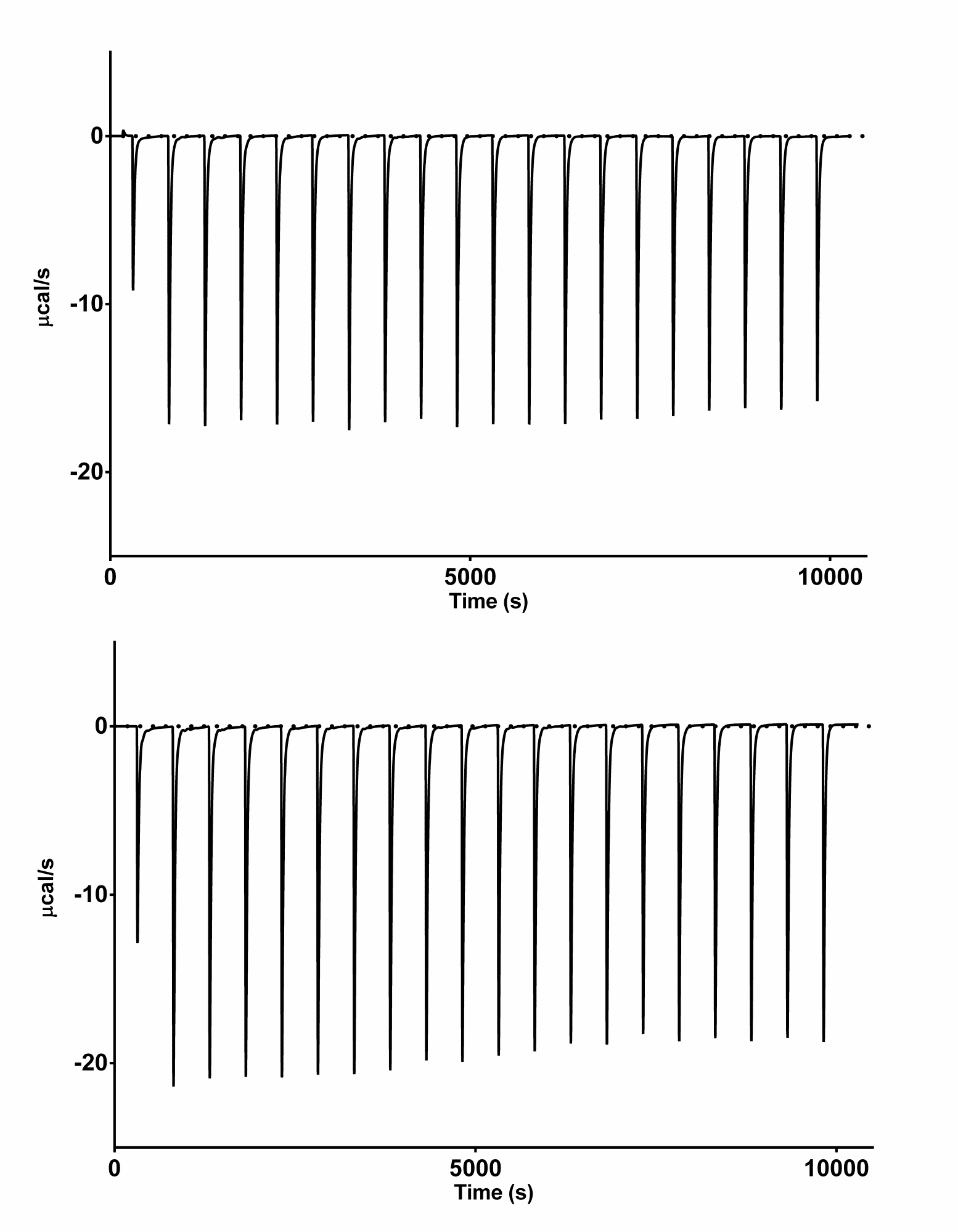


**Fig. S5.** Thermograms obtained by ITC of the titration of choline-*O*-phosphate (300 mM) (2x 5 µL and then 18x 10 µL) into the calorimetric cell (A) with only buffer, or (B) with a 41.6 µM COS solution. No hydrolysis was detected in any experiment. The peaks correspond to the dilution of the choline-*O*-phosphate when it reaches the calorimetric cell. Conditions: 200 mM TRIS-HCl; 500 mM NaCl and 25 °C.


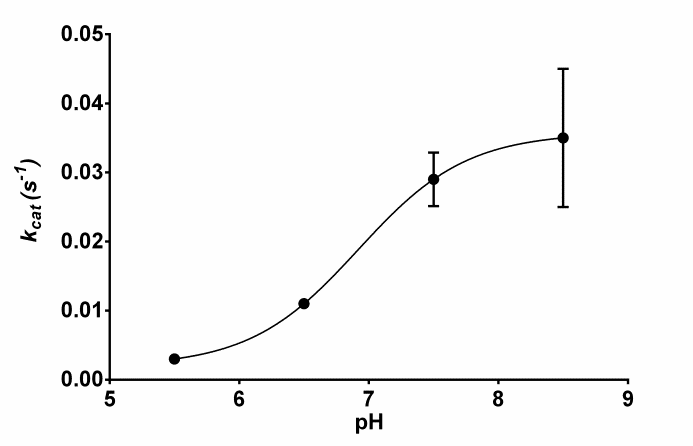


**Fig. S6**. *k_cat_* versus pH at 25 °C for the COS catalyzed pNPS hydrolysis. The large error bars at pH 8.5 are due to the poor fit of data because the substrate solubility does not reach complete enzyme saturation at this pH.


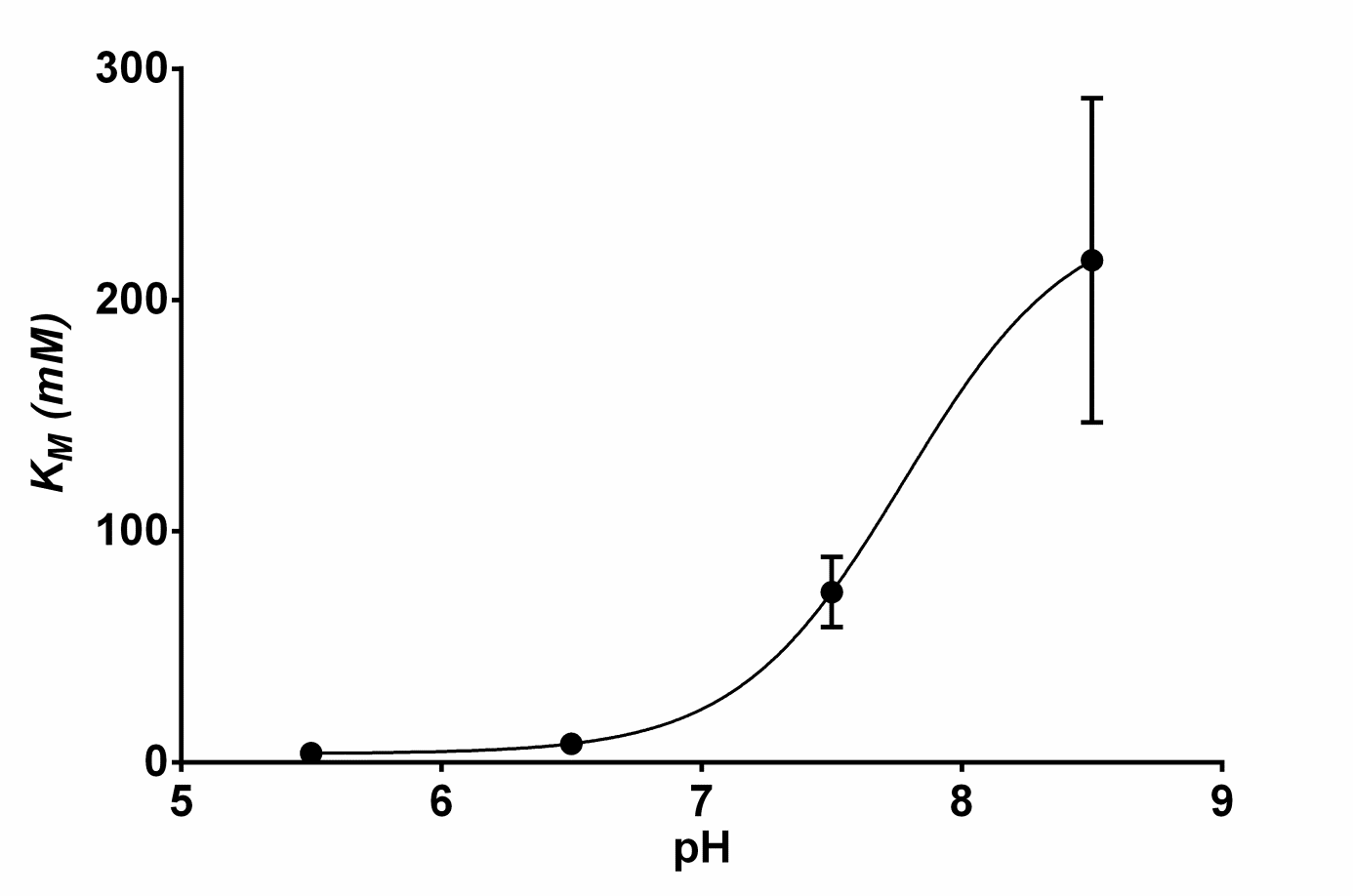


**Fig. S7**. *K_M_* versus pH at 25 °C for the COS catalyzed pNPS hydrolysis. The large error bars at pH 8.5 are due to the poor fit of data because the substrate solubility does not reach complete enzyme saturation at this pH.
